# Role of C/EBP-α in Adriamycin-induced podocyte injury

**DOI:** 10.1038/srep33520

**Published:** 2016-09-20

**Authors:** Fang Zhong, Weiming Wang, Kyung Lee, John Cijiang He, Nan Chen

**Affiliations:** 1 Institute of Nephrology, Shanghai Jiaotong University School of Medicine, Shanghai, China; 2 Division of Nephrology, Department of Medicine, Icahn School of Medicine at Mount Sinai, New York, NY, USA

## Abstract

Podocytes are terminally differentiated epithelial cells in the kidney glomeruli that act as a key component of the glomerular filtration barrier. Although the inciting injury to the podocyte may vary between various glomerular diseases, the inevitable consequence of podocyte injury results in their loss, leading to progressive kidney disease. Here, we report that the expression of CCAAT/enhancer binding protein-α (C/EBP-α), a transcription factor known to interact with and activate PPAR-γ and NF-κB, is suppressed in the glomerular cells, particularly in podocytes, in human kidneys with focal segmental glomerulosclerosis. Genetic ablation of C/EBP-α in podocytes resulted in increased proteinuria, increased podocyte foot process effacement, and to decreased podocyte number in the setting of Adriamycin (ADR)-induced nephropathy. Overexpression of C/EBP-α in human podocytes *in vitro* led to an inhibition of MCP-1 and IL-6 expression in response to TNF-α and IL-1β treatments. Conversely, augmented production of MCP-1 and IL-6 was observed in the glomeruli of C/EBP-α knockout mice and was associated increased infiltration of macrophages *in vivo*. Together, our data suggest that C/EBP-α mediates anti-inflammatory effects in podocytes to confer protection against podocyte injury and loss that may contribute to worsening glomerulosclerosis.

Focal segmental glomeruloscelrosis (FSGS) is a heterogeneous disorder that is a common cause of proteinuria leading to end-stage renal disease (ESRD). Irrespective of etiology, podocyte injury underlies all forms of FSGS^1^. Injury to podocyte leads to actin cytoskeleton derangement and loss of structural integrity that results in foot process effacement and podocyte loss, ultimately leading to progressive kidney disease. Consequently, therapies that prevent podocyte injury and loss will have a major clinical impact in our management of glomerular disease including FSGS.

C/EBP-α, a member of the CCAAT/enhancer binding protein (C/EBP) family of transcription factors, is a key regulator of various cellular functions in a wide range of cell types including those of lung, liver, and of the hematopoietic lineage. It has been shown to regulate adipocyte differentiation through the activation of peroxisome proliferator activated receptor-γ (PPAR-γ)^2^ and to mediate hematopoietic cell differentiation through interaction with GATA, AP-1, and NF-κB^3^,^4^,^5^. In addition, C/EBP-α has been shown to mediate liver growth through interaction of p53 pathway^6^. C/EBP-α is also known to interact with and regulate NF-κB activation, a key regulator of renal inflammation and fibrosis^7^. However, its role in renal cells is not well understood. In the context of kidney disease, it has been reported that the expression of C/EBP-α is lost in streptozotocin-induced diabetic kidneys^8^ and that it may have a role in aristolochic acid-induced renal inflammation and fibrosis^9^. Since C/EBP-α interacts with several transcription factors that are important for kidney disease, such as PPAR-γ, p53, and NF-κB, we hypothesized that C/EBP-α may play an important role in the progression of kidney disease.

We found that C/EBP-α is broadly expressed in all cells of the glomeruli with a strong expression in the podocytes in human kidneys. We also found that its expression was significantly suppressed in kidneys of patients with FSGS. In order to assess whether the decreased expression of C/EBP-α in the setting of glomerular injury affected the disease progression, we generated a podocyte-specific knockout of C/EBP-α and challenged them with Adriamycin (ADR)-induced injury, an experimental model of FSGS. ADR resulted in significantly increased glomerular injury in podocyte-specific C/EBP-α knockout mice in comparison to wildytype mice. The worsened kidney disease in podocyte-specific C/EBP-α knockout mice was associated with heightened activation of NF-κB and glomerular production of MCP-1 and IL-6 and with increased number of infiltrating F4/80-positive macrophages in the renal tubulointerstitium. *In vitro*, overexpression of C/EBP-α decreased the expression of MCP-1 and IL-6 in human podocytes treated with TNF-α and IL-1β. These data suggest that C/EBP-α mediates an anti-inflammatory effect in podocytes and confers protection against podocyte loss in glomerular injury.

## Results

### C/EBP-α expression in human kidneys

**Figure 1:**
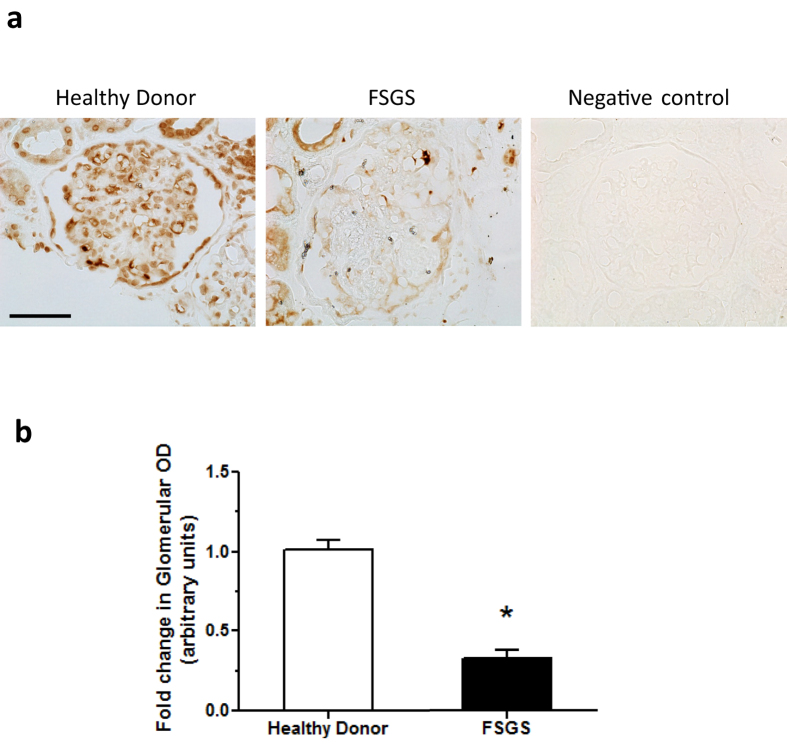
C/EBP-α expression in human kidney. (**a**) Immunohistochemical staining for C/EBP-α performed on healthy donor nephrectomy specimens shows a nuclear distribution in glomerular cells (including endothelial, parietal, and podocytes) and in tubular cells (left). C/EBP-α expression is significantly reduced in kidney biopsy specimens from patients with FSGS (middle) and no staining is detected in no primary antibody negative control (right). Representative images of three subjects in each group are shown (original magnification x400, scale bar: 50 μm). (**b**) Semi-quantification of glomerular C/EBP-α expression in (**a**) is shown. Glomerular region was selected, and optical density (*OD*) was measured and quantified as a relative fold change to healthy donor specimens (*n* = 3, *p < 0.001 vs healthy donor).

We first examined the expression pattern of C/EBP-α in human kidneys. By immunostaining we found that the nuclear expression of C/EBP-α was broadly distributed in all glomerular and tubular cells, with a pronounced expression in the podocytes in normal human kidney tissues obtained from nephrectomy samples (Fig. 1a). Interestingly, the expression of C/EBP-α was significantly reduced in glomerular cells of the kidney from patients with FSGS (Fig. 1b). The quantification of the staining is shown in Fig. 1c. The clinical information of the patients is summarized in Table 1. The decreased expression of C/EBP-α in FSGS patients suggested a potential role of C/EBP-α in worsening of glomerular injury.

### Loss of C/EBP-α in podocytes exacerbated Adriamycin-induced FSGS

**Figure 2:**
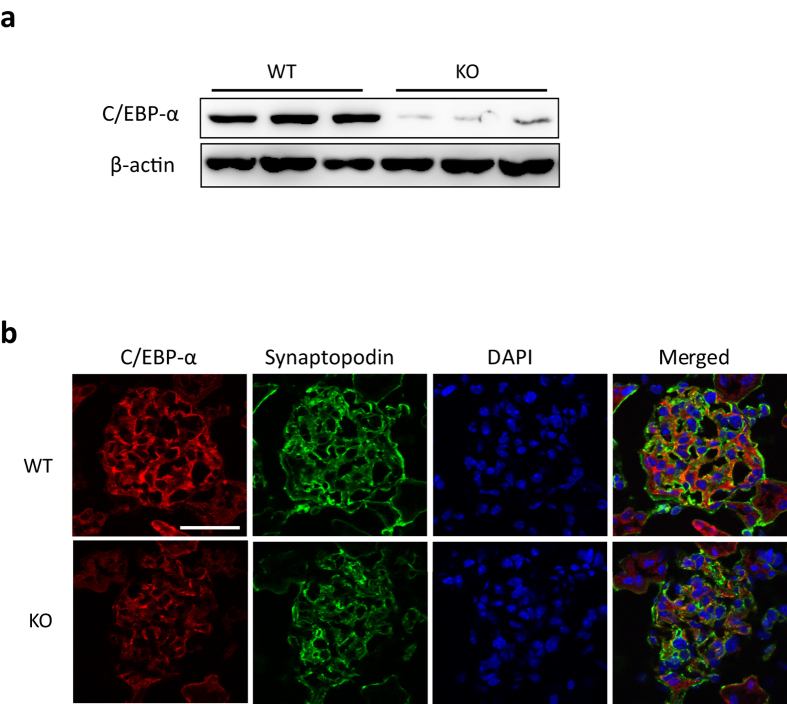
Podocyte-specific knockout of C/EBP-α in mice. (**a**) Western blot analysis of C/EBP-α in the lysates of primary podocytes isolated from WT and KO mice (n = 3 mice in each group). The blots were then stripped and reprobed for β-actin as loading controls. (**b**) Immunostaining of C/EBP-α and podocyte marker synaptopodin shows decreased C/EBP-α in podocytes in KO kidney sections in comparison to WT mice.

Next we sought to determine the role of C/EBP-α *in vivo* in the context of glomerular injury. Since C/EBP-α was highly expressed in podocytes in human kidneys and global *Cebpa* knockout mice are not viable, we developed podocyte-specific *Cebpa* knockout mice by crossing *Cebpa* floxed mice (*Cebpa*^*fl/fl*^) with transgenic mice expressing the Cre recombinase under the podocin promoter, podocin-Cre (Pod-Cre) (both obtained from The Jackson Laboratory). *Cebpa*^*fl/fl*^;*Pod-Cre* mice were viable, fertile, and without discernable defects in phenotype. We confirmed a specific knockout of C/EBP-α in podocytes by western blot analysis of C/EBP-α in primary podocytes from homozygous *Cebpa*^*fl/fl*^;*Pod-Cre* (KO) and control *Cebpa*^+/+^*;Pod-Cre* mice (WT), isolated as described previously^10^ (Fig. 2a). The loss of C/EBP-α in podocytes was further confirmed by double immunostaining of C/EBP-α and podocyte marker, synaptopodin. At baseline KO mice did not develop proteinuria or kidney injury when observed at either 6 months or 12 months of age (data not shown).

**Figure 3:**
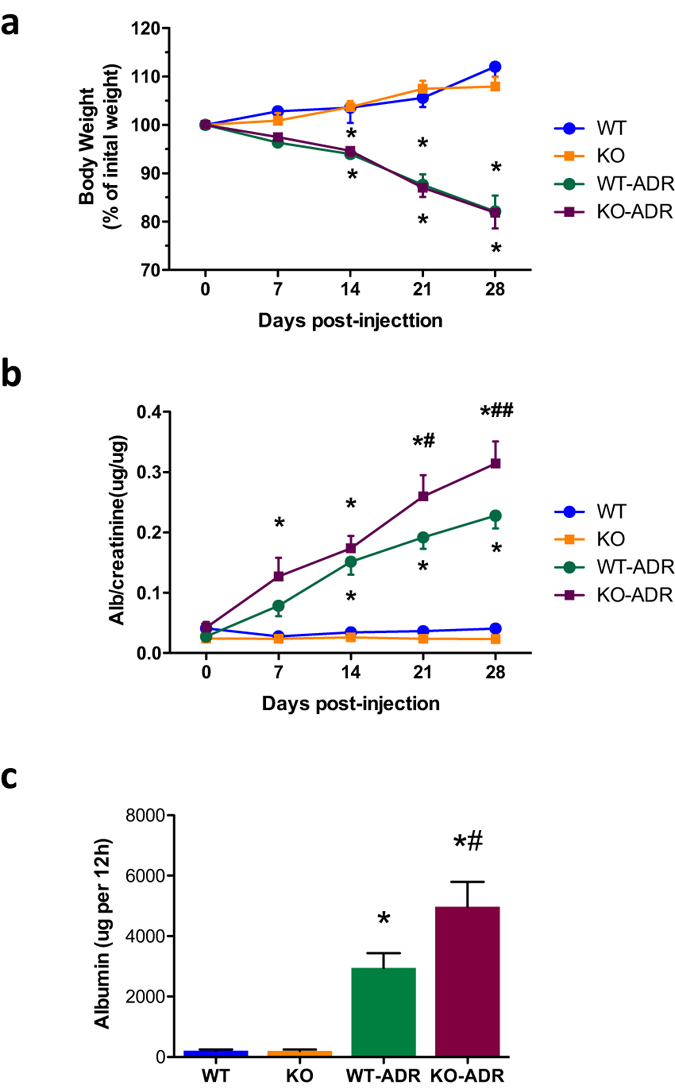
Worsened proteinuria in KO-ADR mice. (**a**) Body weight of mice post-injection of vehicle (WT and KO) or ADR (WT-ADR and KO-ADR) are shown. Both ADR-WT and ADR-KO mice experienced similar weight loss following ADR injection. (**b**) Development of proteinuria in ADR-injected mice was assessed by urinary albumin to creatinine ratio. (**c**) The 12 h urinary albumin excretion at 4 weeks among these mice. (*n* = 6, *p < 0.001 vs WT and KO, ^#^*p* < 0.05 vs WT-ADR).

**Figure 4:**
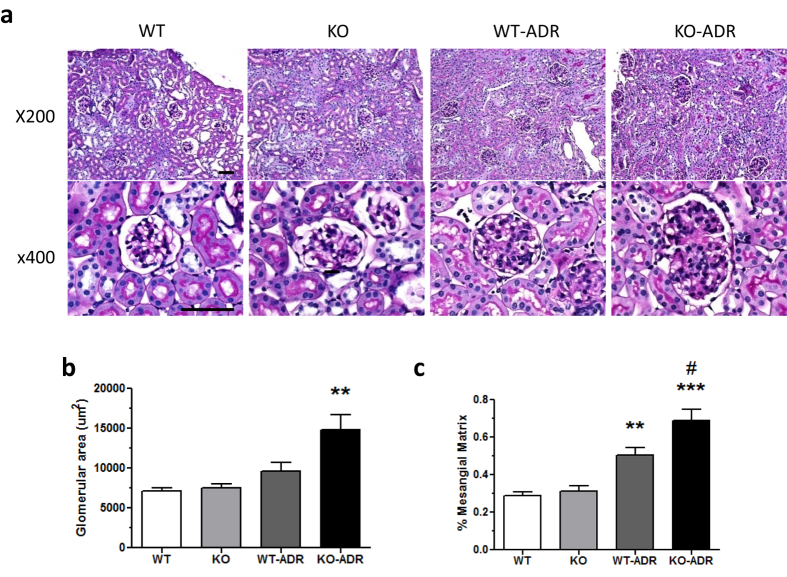
Increased mesangial and glomerular area in KO-ADR mice. (**a**) Representative images of periodic acid–Schiff (PAS)-stained kidneys at 4 weeks post-injection. Glomerular hypertrophy and mesangial area are significantly increased in KO-ADR in comparison to WT-ADR (Original magnification x200 and x400). (**b**) Quantification of glomerular area at 4 weeks post-injection. Values are mean ± SEM (n = 60 glomeruli per group of 6 mice, **p < 0.01 vs other groups). (**c**) Quantification of area of mesangial fraction in the glomeruli at 4 weeks post-injection (*n* = 6, **p < 0.01 and ***p < 0.001 vs WT, ^#^*p* < 0.05 vs WT-ADR).

In order to assess the role of C/EBP-α, glomerular injury was induced in 8-week old WT and KO by intravenous injection of ADR (18 mg/kg). Saline-injected mice were used as controls and all mice were sacrificed 4 weeks post-injection. As expected, both WT and KO mice experienced similar weight loss following ADR injection (WT-ADR and KO-ADR) as compared to saline-injected controls (WT and KO) (Fig. 3a). Assessment of total renal function with blood urea nitrogen (BUN) analysis also showed similarly elevated BUN in ADR-injected mice in comparison to vehicle controls (Supplementary Figure 1). However, we observed a significant increase in albuminuria in KO-ADR mice at 7 days post injection compared to saline-injected controls, whereas significant increase was not observed in WT-ADR mice until 14 days post injection (Fig. 3b). In addition, starting from 21 days post ADR injection, KO-ADR mice exhibited a significantly higher albuminuria than WT-ADR. The increase in albuminuria in KO-ADR mice was further confirmed by measuring the total albumin level in a 12-hour urine collection at 4 weeks after ADR injection (Fig. 3c). Morphometric analysis of kidney histology revealed an increase in glomerular volume and mesangial matrix area in both WT-ADR and KO-ADR kidneys as compared with saline-injected control kidneys (Fig. 4a–c). However, both glomerular volume and fraction of mesangial matrix area were significantly higher in KO-ADR mice than in WT-ADR mice (Fig. 4a–c).

**Figure 5:**
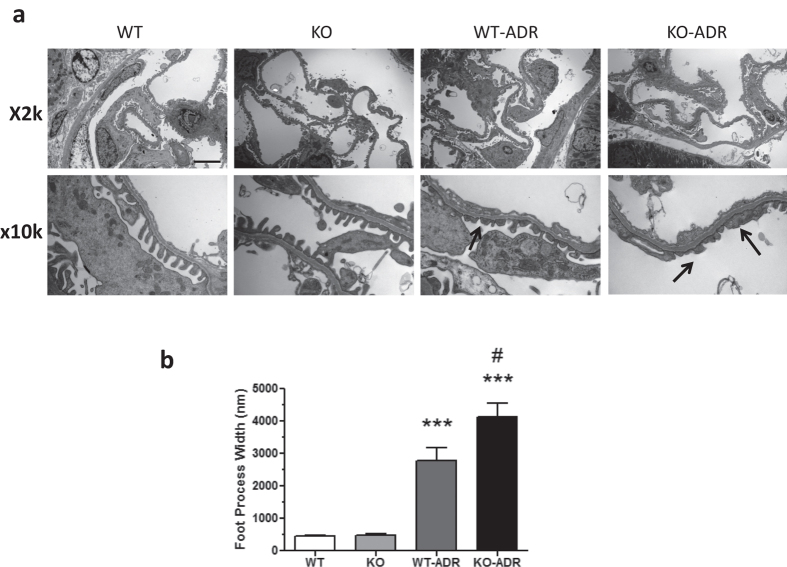
Increased podocyte foot process effacement in KO-ADR mice. (**a**) Representative electron micrograph from WT, KO, WT-ADR, and KO-ADR mice are shown with both low (x2K) and high (x10K) magnifications (scale bar: 5 μm). Arrows point to examples of effaced foot processes in ADR-injected mice. (**b**) Quantification of average foot process width in each group of mice is shown. Values are mean ± SEM (n = 60 glomeruli per group of 6 mice, ***p < 0.01 vs WT an KO, ^#^*p* < 0.05 vs WT-ADR).

**Figure 6:**
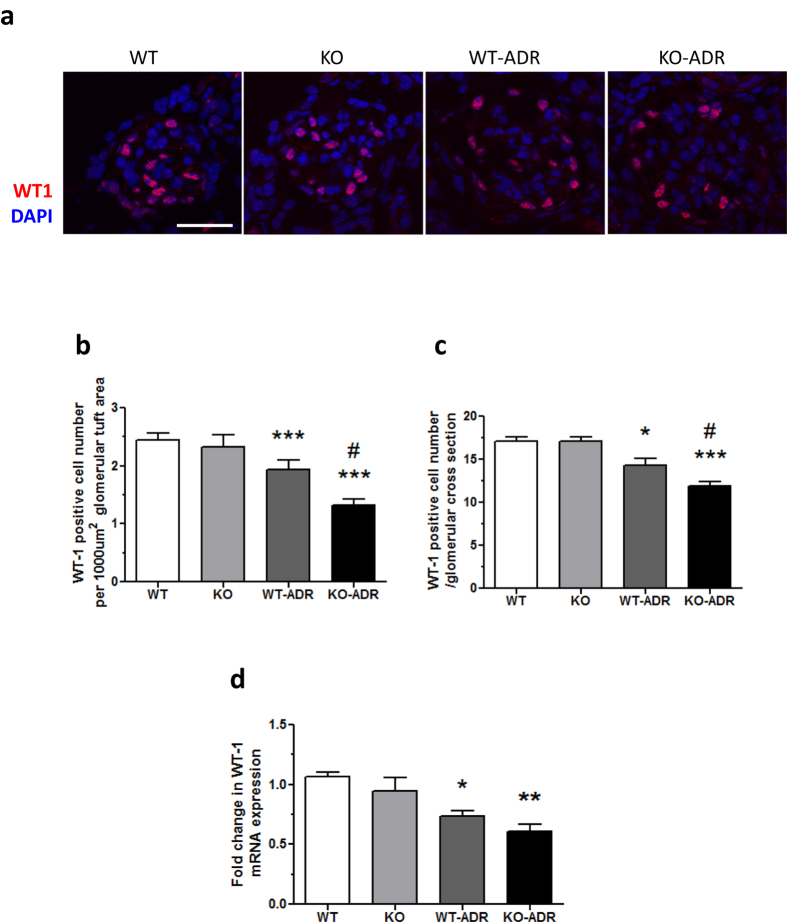
Increased podocyte loss in KO-ADR mice. (**a**) Representative images of WT1 (red) immunostaining and DAPI (blue) of glomeruli are shown (n = 6, original magnification x400, scale bar: 50 μm). (**b, c**) WT1-positive cell number per 1000 µm2 glomerular tuft area (**b**) and WT1-positive cell number/glomerular cross-section (**c**) were quantified and expressed as relative fold changes to WT mice. (**d**) mRNA levels of Wt1 mRNA were measured using real-time PCR. (60 glomeruli per group; n = 6, *p < 0.05, **p < 0.01, ***p < 0.001 vs WT and KO, ^#^*p* < 0.05 compared to WT-ADR).

**Figure 7:**
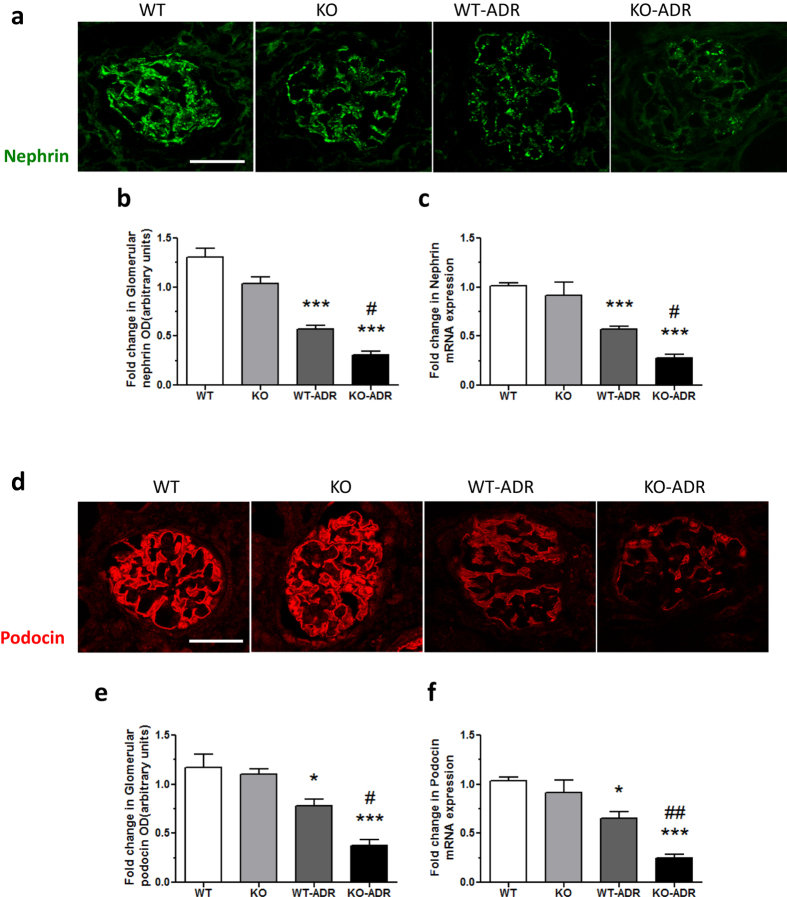
Expression of nephrin and podocin is reduced in KO-ADR mice. (**a,d**) Representative images of immunostaining of nephrin (green, **a**) or podocin (red, **d**) in glomeruli are shown. (**b,e**) Semi-quantification of nephrin or podocin expression is shown. (**c,f**) mRNA levels of nephrin or podocin were measured using real-time PCR. (60 glomeruli per group; n = 6, ***p < 0.001 vs WT and KO, ^#^*p* < 0.05 WT-ADR, original magnification x400, scale bar: 50 μm).

Since KO-ADR mice developed increased albuminuria than WT-ADR mice, we speculated that they might also develop more podocyte injury and loss. Electron microscopy of kidney sections indeed revealed a significant increase in podocyte effacement in the KO-ADR mice compared to WT-ADR mice (Fig. 5a,b). We then assessed the podocyte number per glomerular cross section or per 1000 μm2 of glomerular areas using a known podocyte marker Wilms’ tumor-1 (WT1). As expected, the number of WT1-positive cells were significantly reduced following ADR treatment in both WT and KO mice (Fig. 6a–c). However their reduction were markedly enhanced in KO-ADR glomeruli in comparison to those of WT-ADR. Glomerular Wt1 mRNA expression levels also mirrored these observations (Fig. 6d). Consistent with greater podocyte loss, the expression of podocyte-specific genes nephrin and podocin was also significantly reduced in KO-ADR mice compared to WT-ADR, as determined by real-time PCR analysis of isolated glomeruli and immunofluorescence staining of kidney sections (Fig. 7). Taken together, these data suggest that loss of C/EBP-α in podocytes increases the susceptibility to podocyte injury and loss, resulting in worsened albuminuria and disease progression.

### Overexpression of C/EBP-α inhibited cytokine production in TNF-α or IL-1β stimulated podocytes

**Figure 8:**
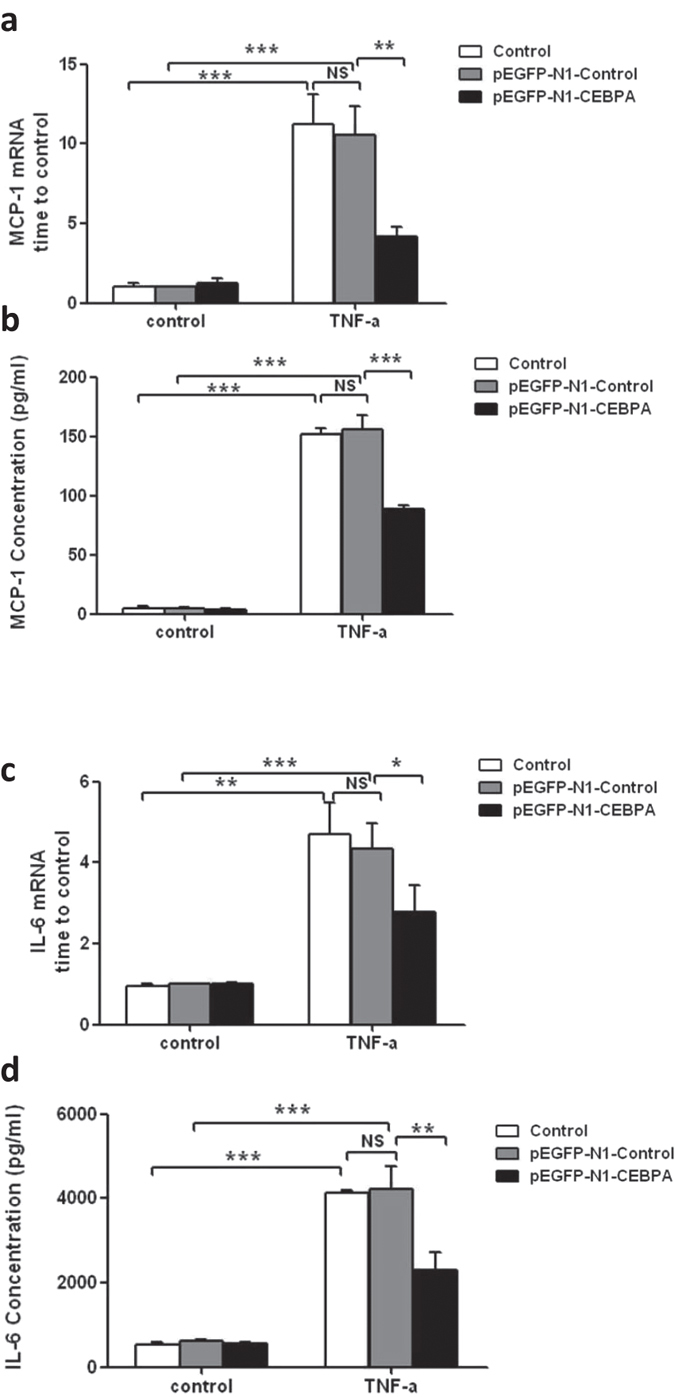
Effect of C/EBP-α overexpression on MCP-1 and IL-6 Expression in podocytes treated with TNF-α. (**a**) MCP-1 mRNA expression in TNF-α-stimulated podocytes. (**b**) MCP-1 production in TNF-α-stimulated podocytes. (**c**) IL-6 mRNA expression in TNF-α-stimulated podocytes. (**d**) IL-6 production in TNF-α-stimulated podocytes. (*p < 0.05,**p < 0.01,***p < 0.001).

**Figure 9:**
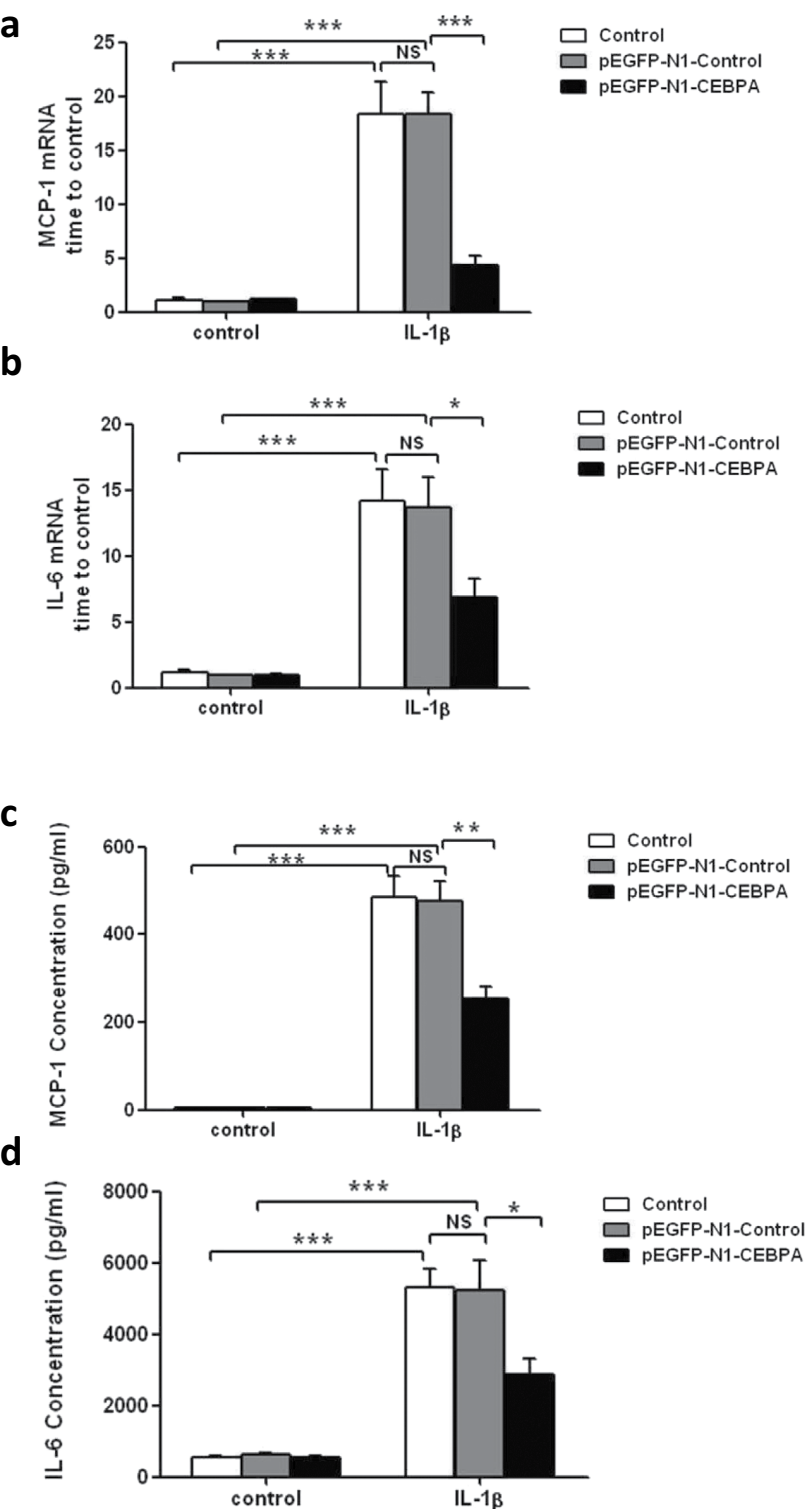
Effect of C/EBP-α overexpression on MCP-1 and IL-6 Expression in podocytes treated with IL-1β. (**a**) MCP-1 mRNA expression in IL-1β-stimulated podocytes; (**b**) MCP-1 production in IL-1β-stimulated podocytes (**c**) IL-6 mRNA expression in IL-1β-stimulated podocytes (**d**) IL-6 production in IL-1β-stimulated podocytes. (*p < 0.05,**p < 0.01,***p < 0.001).

Since the loss of C/EBP-α in podocytes *in vivo* led to greater podocyte injury and aggravated kidney disease following ADR injection, we then sought to determine whether the elevated expression of C/EBP-α would be protective in podocyte injury. We transiently transfected human podocytes with an expression plasmid encoding C/EBP-α fused to a green fluorescent protein (pEGFP-N1-CEBPA) or with a control plasmid (pEGFP-N1). In pEGFP-N1-CEBPA transfected cells, the expression of GFP was found in the nucleus, indicating a nuclear localization of C/EBP-α fusion protein, while the expression of GFP was widely distributed mostly in the cytoplasm of control plasmid-transfected cells (Supplementary Figure 2A). The transfection efficiency was about 70–80%, as assessed by the number of GFP-expressing cells. The expression level and nuclear localization of C/EBP-α were also confirmed by western blot analysis (Supplementary Figure 2B). Since C/EBP-α is known to interact with and regulate NF-κB activation, we investigated whether overexpression of C/EBP-α in podocytes affected NF-κB-mediated cytokine expression and inflammatory response. Transfected podocytes were treated with TNF-α or IL-1β and the level of MCP-1 and IL-6 production was assessed. Indeed, we found that overexpression of C/EBP-α in podocytes attenuated TNF-α-induced MCP-1 and IL-6 expression at both mRNA and protein (Fig. 8). Similarly, overexpression of C/EBP-α in podocytes also inhibited IL-1β-induced MCP-1 and IL-6 expression at both mRNA and protein levels (Fig. 9), suggesting that increased C/EBP-α may protect against podocyte injury through decreasing cytokine production and thus dampening the inflammatory response.

### Loss of C/EBP-α in podocytes resulted in increased glomerular cytokine production and presence of infiltrating macrophages in KO-ADR kidneys

**Figure 10:**
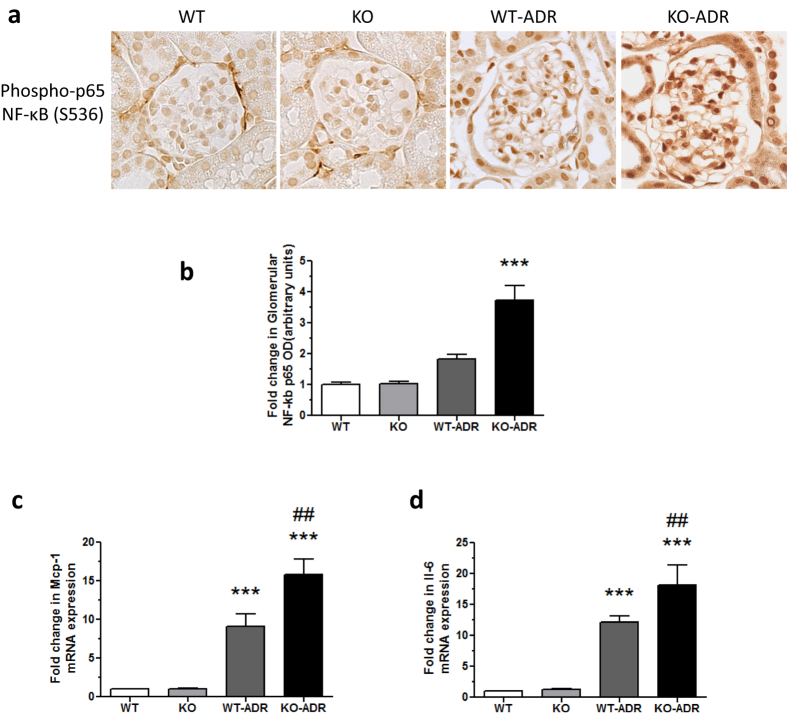
Increased activation of NF-κB and production of MCP-1 and IL-6 in KO-ADR glomeruli. (**a**) Representative images of phosphorylated p65 NF-κB (Ser536) immunostaining are shown. (**b**) Semi-quantification of phospho-p65 NF-κB immunostaining is shown as relative fold change in optical density (OD). (**c,d**) mRNA levels of MCP-1 (**C**) and IL-6 (**d**) in isolated glomeruli from each group of mice were measured using real-time PCR (60 glomeruli per group; n = 6, ***p < 0.001 vs WT and KO, ^##^*p* < 0.01 vs WT-ADR).

**Figure 11:**
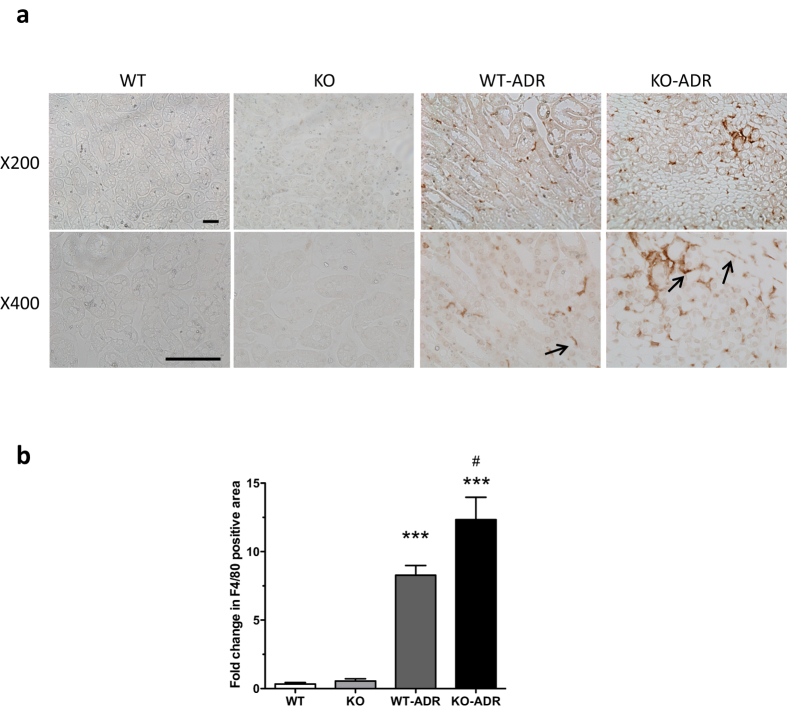
Increased infiltrating macrophages in KO-ADR kidneys. (**a**) Representative images of immunostaining for macrophage marker F4/80 in WT, KO, WT-ADR, and KO-ADR mice at 4 weeks after ADR injection at x200 and x400 magnifcation. (**b**) Quantification of F4/80-positive area between ADR mice and non-ADR mice is shown as fold change relative to WT and KO (*n* = 6, ***p < 0.001 vs WT and KO, ^#^*p* < 0.05 vs WT-ADR).

Since increased C/EBP-α expression decreased the cytokine production *in vitro*, we then examined whether its decreased expression *in vivo* was associated with increased cytokine production and renal inflammation. Since C/EBP-α is known to interact with and regulate NF-κB activation, we first compared the level of NF-κB activation in kidneys of vehicle- and ADR-injected WT and KO mice. As expected ADR-injection led to an increased level of Ser536 phosphorylated NF-κB compared to vehicle at 4 weeks post-injection (Fig. 10a,b), and this increase was much more pronounced in KO-ADR glomeruli compared to those of WT-ADR. Consistent with the increased NF-κB activation in KO-ADR glomeruli, mRNA expression of MCP-1 and IL-6 were also markedly increased in isolated glomeruli of KO-ADR (Fig. 10c,d). In addition, we observed that the increased production of cytokine in KO-ADR was associated with increased presence of infiltrating macrophages in KO-ADR kidneys, as detected by F4/80 immunostaining (Fig. 11). Collectively, our data shows that podocyte-specific loss of C/EBP-α leads to increased podocyte injury and loss, heightened proteinuria, and increased expression of cytokines and infiltrating macrophages in the setting of ADR-induced nephropathy.

## Discussion

In the current study, we found that a significantly decreased glomerular expression C/EBP-α was associated FSGS human kidneys, particularly in podocytes. We found that the podocyte-specific loss of C/EBP-α led to aggravated proteinuria and glomerular injury in the setting of ADR-induced nephropathy, which was associated with increased glomerular MCP-1 and IL-6 production and increased the number of infiltrating macrophages within the tubulo-interstitium. Further, overexpression of C/EBP-α in human podocytes diminished the expression of MCP-1 and IL-6 expression induced by TNF-α and IL-1β treatment *in vitro*, further confirming the protective role of C/EBP-α in the inflammatory response in podocytes.

It has been shown that the expression of chemokines is closely associated with the inflammation in the diseased kidney^11^,^12^. Both intrinsic renal cells and the inflammatory cell can express chemokines, and chemokine secretion can further amplify the inflammatory response^13^. MCP-1 and IL-6 are two important chemokine and cytokine that are increased in the diseased kidney and are highly associated with renal interstitial inflammation^14^,^15^,^16^. Our data suggest C/EBP-α exerts anti-inflammatory effects through the inhibition of MCP-1 and IL-6 expression in podocytes, which may be the basis of the observed renorotective effects of C/EBP-α against the progression of ADR-induced nephropathy in mice.

While the exact mechanism of how C/EBP-α prevents kidney disease progression is not entirely clear, our data suggest that C/EBP-α may have significant anti-inflammatory effects in podocytes through the inhibition of cytokine production. This is likely achieved through inhibition of NF-κB activation by direct interaction as shown in the previous studies^7^. NF-κB has been shown to play a major role in the progression of kidney disease^17^ and inhibition of this pathway may have a significant renoprotective effect^18^. Indeed, our recent work suggests that NF-κB activation mediates podocyte injury in diabetic nephropathy^19^. C/EBP-α is also known to interact with PPAR-γ pathway^2^, which also has been shown to be protective against podocyte injury by inhibition of inflammatory pathways^20^. C/EBP-α also interacts with p53 pathway^6^ and p53 is a key mediator of podocyte apoptosis in kidney disease^21^,^22^. It remains to be determined whether C/EBP-α also interacts with other transcription factors to improve renal injury in the setting of FSGS. Since our study is limited to ADR-induced glomerular injury involving cellular toxicity, current findings will be corroborated using a second genetic model of podocyte injury in future studies.

In summary, we report here that C/EBP-α is highly expressed in glomerular cells, particularly in podocytes, and that the loss of glomerular C/EBP-α expression is associated with FSGS in human patients. Podocyte-specific knockout of C/EBP-α leads to more severe kidney disease in the setting of ADR-induced nephropathy, suggesting an important role of C/EBP-α in protection of podocytes against injury and loss.

## Experimental Procedures

### Immunohistochemistry on human kidney samples

Archived kidney biopsies (n = 15) with diagnosis of FSGS were obtained according to the approved protocol by the Institutional Review Board for Clinical Study at Ruijin Hospital, Shanghai Jiaotong University School of Medicine. All experimental methods were performed in accordance with the approved guidelines. Specimens were initially baked for 20 minutes in 55–60 °C oven and then processed as described previously below. Briefly formalin-fixed and paraffin-embedded sections were deparaffinized, and endogenous peroxidase was inactivated with H_2_O_2_. Sections were then blocked in 2% goat serum in phosphate-buffered saline (PBS) for 1 hour at room temperature and then incubated with a rabbit anti-C/EBP-α antibody (sc-30106, Santa Cruz) at 4 °C overnight. The next day, sections were washed three times with PBS and then incubated with secondary antibody for 30 minutes. Positive staining was revealed by peroxidase-labeled streptavidin and diaminobenzidine substrate. The control included a section stained with only secondary antibody.

### Generation of podocyte-specific CEBP-α knockout mouse model

Animal studies were performed in accordance with the approved protocol and guidelines of Institutional Animal Care and Use Committee at the Icahn School of Medicine at Mount Sinai (New York, NY). Mice were housed in a specific pathogen-free facility with free access to chow and water and a 12-hour day/night cycle. Breeding and genotyping was done according to standard procedures. Podocin-Cre and floxed *Cebpa* (*Cebpa*^*fl/fl*^) mice in C57BL/6 background were obtained from The Jackson Laboratory (Bar Harbor, ME). Male mice expressing *Cre* with homozygous floxed *Cebpa* alleles were used as the experimental group (Cebpa-KO). Mice with two wildtype *Cebpa* alleles (*Cebpa*^+/+^) and *Cre* expression were used as controls (Cebpa-WT). Genotyping by tail preparation and PCR were performed at 3 weeks of age as previously described^23^.

### ADR murine model

In the ADR model, Cebpa-WT and –KO mice (12 weeks of age) were administered ADR (18 mg/kg) intravenously by tail vein injection^24^. Urine was collected weekly to assess for albuminuria, and mice were sacrificed 4 weeks after the treatment. As demonstrated by previous studies^25^ there is typically mesangial expansion, tubular vacuolization, and mild interstitial proliferation at 3 weeks after ADR treatment. Significant glomerulosclerosis and tubulointerstitial inflammation were not observed until week 4. Since our study was performed on a resistant mouse strain (C57BL/6J) and we observed a significant increase in albuminuria at 4 weeks, the mice were sacrificed at this time point to determine the extent of kidney injury.

### Measurement of urine albumin and creatinine

Urine albumin was quantified by ELISA using a kit from Bethyl Laboratories, Inc. (Houston, TX). Urine creatinine levels were measured in the same samples using QuantiChrom^TM^ creatinine assay kit (DICT-500) (BioAssay Systems) according to the manufacturer’s instruction. The urine albumin excretion rate was expressed as the ratio of albumin to creatinine. 12-hour urine collections in the metabolic cages were also used for determination of urinary albumin excretion.

### Measurement of BUN

Blood urea nitrogen (BUN) was measured by using a commercially available kit (Bioassay Systems, Hayward, CA) according to manufacturer’s protocol.

### Kidney histology

Kidneys were removed and fixed with 4% paraformaldehyde for 16 hours at 4 °C. The 4μm sections were cut from paraffin-embedded kidney tissues. Sections were stained with periodic acid–Schiff for histology analysis. Assessment of the mesangial and glomerular cross-sectional areas was performed by pixel counts on a minimum of 10 glomeruli per section in a blinded fashion, under 400× magnification (Zeiss AX10 microscope, Carl Zeiss Canada Ltd, Toronto, ON, Canada).

### Isolation of glomeruli from mice for RNA extraction

Mouse glomeruli were isolated as described^26^. Briefly, animals were perfused with Hanks’ buffered salt solution containing 2.5 mg/ml iron oxide and 1% bovine serum albumin. At the end of perfusion, kidneys were removed, decapsulated, minced into 1 mm^3^ pieces, and digested in Hanks’ buffered salt solution containing 1 mg/ml collagenase A and 100units/ml deoxyribonuclease I. Digested tissue was then passed through a 100 μm cell strainer and collected by centrifugation. The pellet was resuspended in 2 ml of Hanks’ buffered salt solution, and glomeruli were collected using a magnet. The purity of glomerular was verified under microscopy. Total RNA was isolated from kidney glomeruli of mice using TRIzol (Invitrogen).

### Isolation of primary podocytes

Briefly, isolated glomeruli were transferred onto a 6-cm tissue culture dish coated with type I collagen and cultured in RPMI 1640 medium (Life Technologies) supplemented with 10% fetal bovine serum and 1% penicillin/streptomycin. DOX was added to the culture medium to achieve a final concentration of 2 μg/mL, where specified. Glomeruli and cells were allowed to attach to the plate for 5 days in a 37 °C incubator without any agitation. Five days later, glomeruli and outgrowth cells were detached from the plate using a 0.12% trypsin-EDTA solution. Trypsinized cells [primary glomerular epithelial cells (PGECs)] were strained using a 40-μm cell strainer and replated onto collagen-coated dishes. PGECs were allowed to grow to 80% confluence before passaging at a ratio of 1:3. PGECs are cells derived from decapsulated glomeruli, isolated by perfusion of magnetic particles. PGECs consist mostly of podocytes, with approximately 70% of PGECs being positive for podocalyxin staining.

### Podocyte transfection and treatment

Conditionally immortalized human podocytes (AB8/13) were propagated at 33 °C in RPMI 1640 medium with 10% fetal calf serum (FCS) and insulin, transferrin and sodium selenite (ITS) media supplement (Sigma-Aldrich, St. Louis, MO, USA), and then shifted to 37 °C for 1 week to induce differentiation^27^. Podocytes were transfected with either pEGFP-N1 control plasmid or with pEGFP-N1-CEBPA. Post 24 hours transfection, the media was changed and cell were stimulated with either TNF-α (10 ng/ml) and IL-1β (10 ng/ml). Cells were harvested at 0 h, 4 h, 8 h, 16 h post stimulation and proteins were extracted using a standard protocol. The experiment was repeated three times. pMK-CEBPA plasmid was purchased by Invitrogen Corporation; pEGFP-N1 plasmid by Clontech. The Lipofectamine 2000 (Invitrogen, Carlsbad, CA) was used for transfection.

### Western Blot

Cells were homogenized in lysis buffer containing protease inhibitor cocktail. Equal amounts of protein samples were electrophoretically separated on SDS polyacrylamide gel, transferred to PVDF membranes (Millipore) and probed with primary antibodies Membranes were then washed with PBST and incubated with a secondary antibody (horseradish peroxidase conjugated antibodies to mouse IgG or to rabbit IgG). Blots were developed with the enhanced chemiluminescence system. Densitometry analysis for quantification was performed as described previously^28^.

### Real-time PCR

Total RNA was extracted by using TRIzol (Invitrogen). First strand cDNA was prepared from total RNA (2.0 μg) using the Superscript ^TM^ III first strand synthesis kit (Invitrogen), and cDNA (1 μl) was amplified in triplicate using SYBR GreenER qPCR Supermix on an ABI PRISM 7900HT (Applied Biosystems, Foster City, CA). The primer sequences are listed in Supplementary Table 1. Light Cycler analysis software was used to determine crossing points using the second derivative method. Data were normalized to housekeeping genes (GAPDH) and presented as fold increase compared with RNA isolated from control wildtype animals using the 2-∆∆^*CT*^ method.

### Immunofluorescence

Kidney sections from these mice were prepared in an identical fashion. Immunostaining was performed using rabbit anti-podocalyxin (R&D Systems), rabbit anti-nephrin (a gift from Dr. Larry Holzman), and mouse anti-WT1 antibodies (Santa Cruz Biotechnology). After washing, sections were incubated with a fluorophore-linked secondary antibody (Alexa Fluor 488 anti-rabbit IgG and Alexa Fluor 568 anti-mouse IgG from Invitrogen). Slides were subsequently mounted in Aqua Poly/Mount (Polysciences Inc.) and images were acquired using AxioVision IIe microscope with a digital camera.

### Estimation of Glomerular Volume and Mesangial Area

Quantification of mesangial area and glomerular volume was performed as previously described^29^. In brief, digitized images were scanned and profile areas were traced using ImageJ 1.26t software. Mean glomerular tuft volume (GV) was determined from mean glomerular cross-sectional area (GA) by light microscopy. GA was calculated based on average area of 30 glomeruli in each group and GV was calculated based on the following equation:


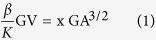


where *β* = 1.38, the shape coefficient of spheres (the idealized shape of glomeruli), and *κ* = 1.1, the size distribution coefficient.

Mesangial expansion was defined as periodic acid-Schiff-positive and nuclei-free area in the mesangium. Quantification of mesangial expansion was based on at least 60 glomeruli cut at the vascular pole in each group.

### Quantification of immunostaining

After sections were stained with anti-C/EBP-α antibody, negatives were digitized, and images with a final magnitude of approximately x400 were obtained. ImageJ 1.26t software was used to measure the level of immunostaining in the glomeruli. First, the images were converted to 8-bit grayscale. Next, the glomerular region was selected for measurement of area and integrated density. Next, the background intensity was measured by selecting three distinct areas in the background with no staining. The corrected optical density (COD) was determined as shown below:


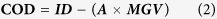


where ID is the integrated density of the selected glomerular region, A is the area of the selected glomerular region, and MGV is the mean gray value of the background readings)^30^. An average of at least 60 glomeruli were measured in each group in a blinded fashion.

### Electron Microscopy

Tissues were fixed in 2.5% glutaraldehyde with 0.1M sodium cacodylate (pH 7.4) for 72 hr at 40 °C. Samples were further incubated with 2% osmium tetroxide and 0.1M sodium cacodylate (pH 7.4) for 1 hr at 40 °C. Ultrathin sections were stained with lead citrate and uranyl acetate and were viewed on a Hitachi H7650 microscope. Briefly, negatives were digitized, and images with a final magnitude of approximately X10,000 were obtained. ImageJ 1.26t software (National Institutes of Health, rsb.info.nih.gov) was used to measure the length of the peripheral GBM, and the number of slit pores overlying this GBM length was counted. The arithmetic mean of the foot process width (*W*_FP_) was calculated as shown below:


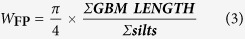


where Σslits indicates the total number of slits counted; ΣGBM LENGTH indicates the total GBM length measured in one glomerulus, and π/4 is the correction factor for the random orientation by which the foot processes were sectioned^31^.

### Statistical analysis

Data were expressed as mean ± SEM. The unpaired t test was used to analyze data between two groups. The analysis of variance followed by Bonferroni correction was used when more than two groups were present. All experiments were repeated at least three times, and representative experiments are shown. Statistical significance will be considered when p < 0.05.

## Additional Information

**How to cite this article**: Zhong, F. *et al*. Role of C/EBP-α in Adriamycin-induced podocyte injury. *Sci. Rep*. **6**, 33520; doi: 10.1038/srep33520 (2016).

## Supplementary Material

Supplementary Information

## Figures and Tables

**Table 1:** Clinical characteristics of patients.

	SCR (μmol/l)	BUN (mmol/l)	Alb (g/L)	TG (mmol/L)	TC (mmol/L)	Glu (mmol/l)	24hpro (mg)
Normal	76.5 ± 5.50	4.87 ± 1.20	41.9 ± 3.11	0.97 ± 0.61	4.43 ± 0.57	4.29 ± 0.42	<30–300
FSGS	118.9 ± 49.1^*^	6.30 ± 2.03^*^	35.7 ± 8.29^*^	2.21 ± 1.05^*^	5.81 ± 1.65^*^	4.60 ± 0.79	877.1 ± 640.1

Abbreviations: FSGS, focal segmental glomerulosclerosis; Scr, serum creatinine; BUN, blood urea nitrogen; ALB, albumin; TG, Triglycerides; TCH, total cholesterol; Glu, glucose; 24hpro, 24 h urine protein; *p < 0.05 (Tested vs. Normal).
