# Supplementary material for: Role of C/EBP-α in Adriamycin-induced podocyte injury
Source: Sci Rep. 2016 Sep 20;6:33520. doi: 10.1038/srep33520 (PMC5028749; doi:10.1038/srep33520)
Supplement: Supplementary Information [file srep33520-s1.pdf]

Supplementary Figures

Role of C/EBP-α in Adriamycin-induced podocyte injury

Fang Zhong[1, 2], Weiming Wang[1] *, Kyung Lee[2], John Cijiang He[2] * and Nan Chen[1]

[1] Institute of Nephrology, Shanghai Jiaotong University School of Medicine, Shanghai, China
[2] Division of Nephrology, Department of Medicine, Icahn School of Medicine at Mount Sinai, New York, NY

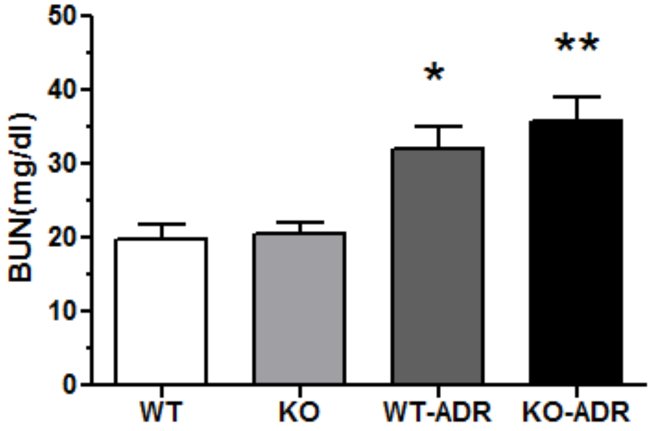

Supplementary Figure 1: Decreasing renal function in WT-ADR and KO-ADR mice
Renal function of mice in each group were assessed using the blood urea nitrogen (BUN) analysis on serum samples obtained 4 weeks post injection of vehicle or ADR (n = 6, *p <0.05, **p <0.01 vs WT and KO).

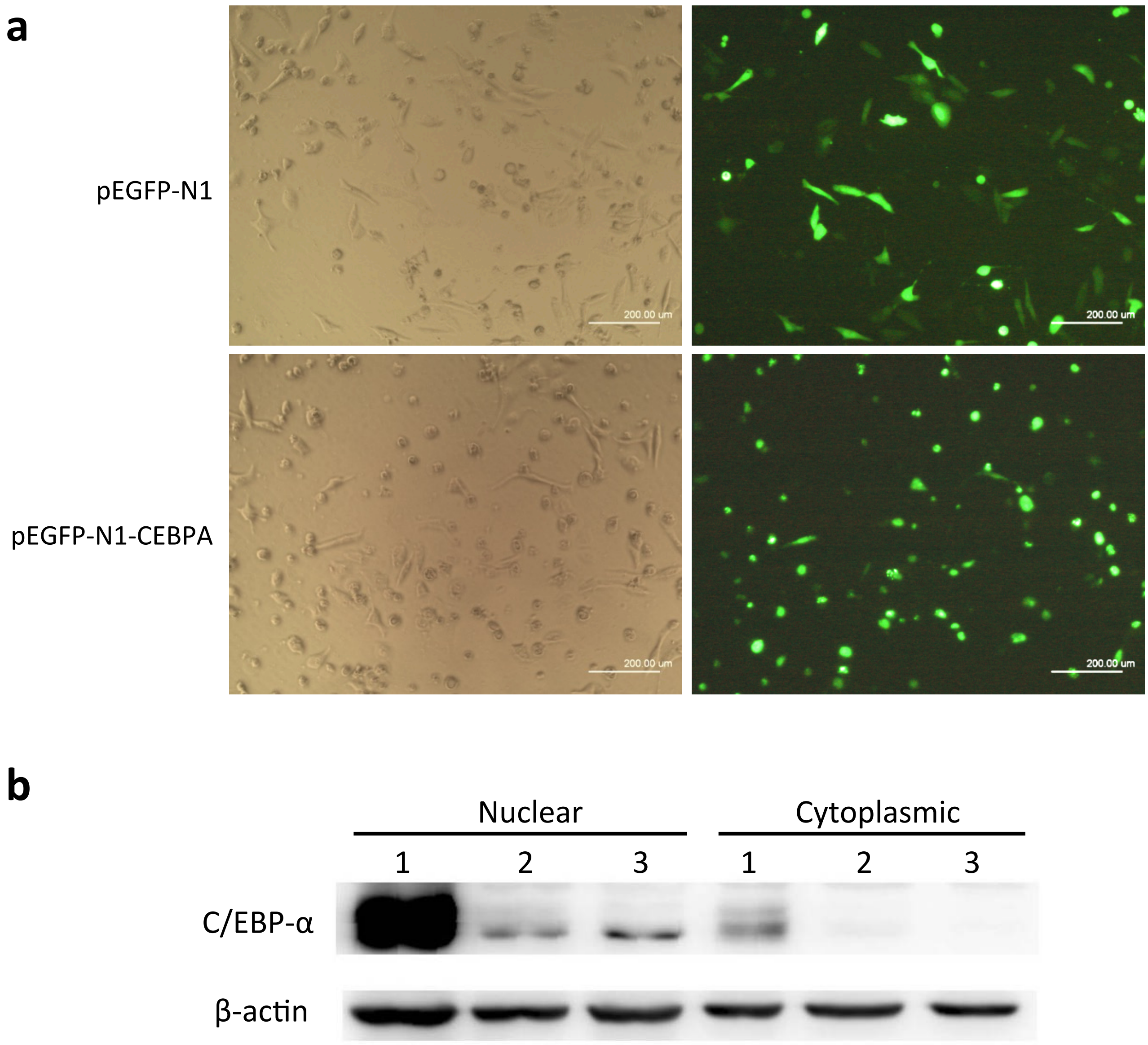

Supplementary Figure 2: Expression of C/EBP-α in human podocytes

(A) 70-80% of enhanced green fluorescent protein (EGFP) expression was confirmed with phase contrast bright-field (left panels) and epi-fluorescence (right panels) microscopy in podocytes transfected with either pEGFP-N1-CEBPA plasmid (Top) or with pEGFP-N1 control plasmid (Bottom). (B) Confirmation of C/EBP-α expression in nucleus and cytoplasm fractions of transfected podocytes by western blot analysis. Analysis of nuclear and cytoplasmic fractions of podocytes expressing C/EBPα-EGFP (1), EGFP (2), or untransfected (3) are shown. β-actin was used as a loading control.

Supplementary Table 1: Primer sequences for real-time PCR

Gene	Forward	Reverse
CEBPA1F (Xho I)	5'-ATCATCCTCGAGATGGAGTCGGCCGACTT-CTACGAGG-3’	5'-ATCATCGGATCCCGCGCAGTTGCCCATG-GCCTTGC-3’
Gapdh	5'-AGCACCAGACCACAGGTGA-3’	5'-GAGGGTGTCGTAGGTGATGC-3’
Cebp-a	5'-TGGCCTGGAGACGCA ATGA-3’	5'-CGCAGAGATTGTGCGTCTTT-3’
Nphs1	5'-GTGCCCTGAAGGACCCTACT-3’	5'-CCTGTGGATCCCTTTGACAT-3’
Nphs2	5'-CTTGGCACATCGATCCCTCA-3’	5'-CGCACTTTGGCCTGTCTTTG-3’
Wt1	5'-GAGAGCCAGCCTACCATCC-3’	5'-GGGTCCTCGTGTTTGAAGGAA-3’
GAPDH	5'-CAGGGCTGCTTTTAACTCTGGTAA-3’	5'- GGGTGGAATCATATTGGAACATGT-3’
MCP-1	5'-CAGCCAGATGCAATCAATGC-3’	5'-GTGGTCCATGGAATCCTGAA-3’
IL-6	5'- GCACCTCAGATTGTTGTTG-3’	5'- AAATAGTGTCCTAACGCTCATAC-3’
